# Persistent Mixed Donor Chimerism following Double Umbilical Cord Transplantation in a Patient with T-Cell Prolymphocytic Leukemia

**DOI:** 10.1155/2019/8437805

**Published:** 2019-09-11

**Authors:** Ronak H. Mistry, Elizabeth O. Hexner, James K. Mangan

**Affiliations:** ^1^Department of Internal Medicine, Pennsylvania Hospital of the University of Pennsylvania, 800 Spruce Street, Philadelphia, PA 19107, USA; ^2^Abramson Cancer Center, Perelman Center for Advanced Medicine, University of Pennsylvania, 34th Street and Civic Center Boulevard, 12th Floor South Pavilion, Philadelphia, PA 19104, USA

## Abstract

T-cell prolymphocytic leukemia (T-PLL) is a rare and aggressive postthymic T-cell neoplasm with an associated survival time of 1 year when left untreated. Current standard of care for T-PLL is with alemtuzumab, followed by allogeneic or autologous stem cell transplant. Little is found in the literature about alternative donor transplantation in T-PLL. Here, we present the case of a patient treated with double umbilical cord blood transplantation, which resulted in initial complete remission. An unusual outcome of this case is that coengraftment of both cords was established. After 16 months, the patient had relapse of the disease, unfortunately, prompting treatment with alemtuzumab and pentostatin, which resulted in remission once again. Here, we report a unique phenomenon whereby single-cord dominance occurred after treatment with these agents, suggesting that anti-T-cell therapy after transplant may help achieve single-unit dominance. A second relapse of the disease occurred six months thereafter, ultimately resulting in the patient's death, highlighting the aggressive nature of this disease.

## 1. Introduction

T-cell prolymphocytic leukemia (T-PLL) is a rare postthymic T-cell neoplasm, accounting for approximately 2% of all leukemia cases, with a greater incidence in males beginning in their sixth decade [1]. Typically, untreated T-PLL is associated with a survival of less than 1 year [2]. The aggressive nature of this disease, and its rarity, presents both diagnostic and therapeutic challenges to physicians [2]. Current standard of care for T-PLL includes the use of alemtuzumab (Campath-1H), a humanized IgG1 antibody specific for the CD52 antigen which is highly expressed on normal and malignant lymphocytes [1, 3]. Thereafter, postremission therapy with either autologous or allogeneic stem cell transplant (SCT) has been associated with disease-free and long-term survival, with a median overall survival of 48 months for both allogeneic and autologous SCT, compared to 20 months for those patients who received alemtuzumab alone [1]. There is a paucity of data on alternative donor transplantation in T-PLL. A Japanese group reported the first case of an umbilical cord blood transplant for T-PLL in 2005 [4]; however, we cannot find subsequent reports of umbilical cord blood transplants for this disease. Accordingly, we report here a second case of umbilical cord transplantation used for T-PLL, employing two umbilical cord units this time. Double umbilical cord blood transplant (dUCBT) is increasingly utilized in adults as it appears to improve outcomes, though almost inevitably only one cord blood unit engrafts, a phenomenon known as single-unit dominance [5]. This case is notable for successful engraftment and complete remission that lasted 16 months despite the presence of residual disease at the time of reduced-intensity transplant. An unusual feature of this case was persistent coengraftment of both umbilical cord units, which can be associated with relapse [6]. Persistent coengraftment is rare in dUCBT and has been reported in a small number of case reports [7]. Following relapse 16 months after transplant, a second remission was achieved with a repeat course of alemtuzumab and pentostatin, which lasted 6 months before relapse occurred again. Unbalanced and dynamic coengraftment endured until frank relapse, when the unit contributing less was no longer detectable. This case illustrates the tenuous nature of complete remissions which involve coengraftment after dUCBT. It also demonstrates that dUCBT can result in complete remissions in T-PLL, though the prognosis of patients remains grim with or without allogeneic SCT. We present details of our case below.

## 2. Case Presentation

A 59-year-old female presented to the local emergency department with reports of fatigue and shortness of breath. The patient was observed to have a WBC >400,000/*μ*L, in addition to pitting edema at the ankles and scattered petechiae. Peripheral smears indicated a marked leukocytosis with atypical blast-like cells. A bone marrow biopsy showed extensive involvement with a T-cell lymphoproliferative disorder with the following immunophenotypes: CD2+, CD4+, CD5+, CD7+, cytoplasmic CD3+, and CD52+, with loss of membrane-associated CD3. Fluorescence in situ hybridization (FISH) indicated a loss of the TCL1 locus, consistent with cyotgenetics showing monosomy 14, as well as numerous other abnormalities (complex and monosomal karyotype), including an addition of 22q. CT imaging indicated splenomegaly and multiple enlarged lymph nodes. Collectively, these findings were diagnostic of T-PLL. This patient was started on intravenous alemtuzumab on a Monday, Wednesday, Friday regimen, initially with a dose escalation of 3 mg on day 1, 10 mg on day 3, and 30 mg on day 5. She continued on 30 mg intravenous alemtuzumab three times weekly for 12 weeks, consistent with current treatment protocols [3].

After four weeks of therapy, the anticipated decrease in WBC was not observed. Therefore, pentostatin was added at 4 mg/m^2^ per week, as previously described [8]. With the addition of pentostatin, bone marrow biopsy indicated complete morphologic and cytogenetic remission. An allogeneic SCT was planned, but since she had neither a related nor a suitable unrelated donor, umbilical cord blood grafts were selected. The grafts were both HLA matched at 5/6 loci. A pretransplant bone marrow biopsy demonstrated a normocellular marrow with trilineage hematopoiesis and 5–10% residual T-PLL cells. Shortly thereafter, the patient underwent reduced intensity dUCBT, conditioned with fludarabine, cyclophosphamide, and total body irradiation [9]; cyclosporine and mycophenolate mofetil were used for graft-versus-host disease (GVHD) prophylaxis. She received 4.2 × 10^7^ total nucleated cells/kg (TNC/kg) from cord 1 (female) and 2.4 × 10^7^ TNC/kg from cord 2 (male). Neutrophil engraftment occurred on day +18, and no signs or symptoms of GVHD ensued. One year after transplantation, a biopsy showed a normocellular marrow (40%) with trilineage hematopoiesis and no overt signs of residual or recurrent T-PLL. Chimerism studies indicated 68% donor 1 and 30% donor 2. A summary of her engraftment analysis is provided in Table 1.

Sixteen months after transplant, she began to experience increased fatigue and developed new leukocytosis. Flow cytometry confirmed a second relapse of the disease. Soon thereafter, retreatment with alemtuzumab and pentostatin began. The patient became profoundly cytopenic following her alemtuzumab and pentostatin therapy. Bone marrow biopsy in mid-October confirmed a third remission, with 99% donor cells, though again with both grafts represented 80% donor 1 and 19% donor 2. She remained in complete remission with incomplete platelet recovery; the percentage of donor 1 increased to 95% and percentage of donor 2 dropped to 5% during this time. Unfortunately, however, after a remission that lasted six months, the patient relapsed for a third time and was refractory to attempts at salvage therapy. She expired after surviving for 29 months following her transplant.

## 3. Discussion

The phenomenon of single-unit dominance has been well described [5]. It is thought to be mediated by rejection of one graft by T cells in the dominant graft [10], though predicting which cord will predominate is not possible [7]. Coengraftment and mixed donor/recipient chimerism have been associated with relapse [6]. Following successful treatment of relapse after dUCBT with alemtuzumab and pentostatin, no recipient chimerism was detectable, while donor 1 engraftment peaked at 95%, but donor 2 still persisted (5%). It is interesting that posttransplant therapy with alemtuzumab and pentostatin brought the patient closer to achieving single-unit dominance, which raises the possibility that anti-T-cell therapy after transplant may promote single-unit dominance and potentially protect against relapse. There is clinical evidence supporting the idea that the presence of specific T-cell populations after transplant can mediate the dominance of one cord blood unit over the other, and preclinical data demonstrating that depletion of immune cell fractions within a graft strongly influences coengraftment [10]. Additional research will be needed to fully elucidate the mechanisms of single-unit dominance after dUCBT and to learn whether immune manipulation in a way that promotes single-unit dominance can reduce relapse risk. This case does illustrate, however, that dUCBT is feasible for T-PLL, and postremission dUCBT should be considered for patients with T-PLL who lack available suitably matched related or unrelated donors. This patient remained relatively responsive to salvage therapies even after relapsing following dUCBT and survived for 12 months following her initial posttransplant relapse and 29 months after transplant. This exceeds the anticipated survival for patients treated with alemtuzumab-based therapies alone without transplant, particularly given detectable residual disease at the time of transplant and treatment with a reduced intensity conditioning regimen.

## Figures and Tables

**Table 1:** Postengraftment analysis following double UCB transplantation, prior to relapse.

Time post-UCB transplant		Donor 1 (female) (%)	Donor 2 (male) (%)
Day +35	Whole blood	48	40
	T-cell subset	>90	<10
Day +141	Whole blood	68	30
	T-cell subset	67	17
Day +251	Whole blood	70	27
	T-cell subset	56	23
12 months	Whole blood	72	21
	T-cell subset	49	44
21 months	Whole blood	95	5
	T-cell subset	79	13
